# Knowledge, attitudes, and practices of Tougan population towards dengue in Burkina Faso in 2024

**DOI:** 10.4102/jphia.v17i1.1451

**Published:** 2026-01-27

**Authors:** Ter Tiero E. Dah, Abdoul S. Moné, Désiré L. Dahourou, Kadari Cissé, Hamidou Savadogo, Nongognan I. Lengane, Zanga D. Ouattara, Abdoulaye Sawadogo, Linda A. Koubi, Isidore T. Traore, Samiratou Ouedraogo, Jocelyne Gare, Abdoulaye H. Diallo, Smaïla Ouedraogo, Blahima Konate, Koiné M. Drabo, Nicolas Meda

**Affiliations:** 1 Department of Public Health, Faculty of Health Sciences Training and Research, University Ledea Bernard Ouedraogo, Ouahigouya, Burkina Faso; 2 Department of Public Health, Faculty of Health Sciences, Training and Research, University Joseph Ki-Zerbo, Ouahigouya, Burkina Faso; 3 Department of Biological Analysis, Tougan Medical Hospital, Tougan, Burkina Faso; 4 National Centre for Scientific and Technological Research, Health Sciences Research Institute, Ouagadougou, Burkina Faso; 5 Regional Medical Hospital of Ziniaré, Ziniaré, Burkina Faso; 6 Department of Public Health, Higher Institute of Health Sciences, University Nazi Boni, Bobo-Dioulasso, Burkina Faso; 7 Department of Public Health Laboratory, Faculty of Health Sciences Training and Research, University Joseph Ki-Zerbo, Ouagadougou, Burkina Faso; 8 National Centre for Scientific and Technological Research, Institute for Society Sciences, Ouagadougou, Burkina Faso

**Keywords:** knowledge, attitudes, practices, dengue, Tougan, Burkina Faso

## Abstract

**Background:**

Recent dengue outbreaks in Burkina Faso have caused substantial morbidity and mortality among semi-urban and urban populations.

**Aim:**

To assess the knowledge, attitudes, and practices (KAP) among the population of Tougan, a semi-urban city, towards dengue.

**Setting:**

Between April 2024 and July 2024, a cross-sectional study was performed among residents and internally displaced populations (IDPs) in Tougan.

**Methods:**

Participants aged 18 years and older, recruited through an adapted three-stage random sampling were eligible. Sociodemographic data as well as information on knowledge, attitudes, and practices (KAP) were collected using a questionnaire. Factors associated with good knowledge of dengue (i.e. knowledge score ≥ 11) were identified using logistic regression models.

**Results:**

A total of 419 participants, including 130 (31.1%) IDPs, were enrolled in the study. The majority were male (53.9%), with a mean age of 38.5 years (standard deviation [s.d.] 12.9). The mean scores of KAP regarding dengue were 10.1 (s.d. 1.2), 13.5 (s.d. 1.9), and 9.4 (s.d. 2.2), respectively. Internally displaced populations had significantly lower levels of knowledge of dengue (adjusted odds ratio [aOR]: 0.49, 95% confidence interval [CI]: 0.29–0.81, *p* = 0.006) compared to residents. Moreover, participants with stagnant water at their home (aOR: 3.03, 95% CI: 1.84–4.96, *p* < 0.001) and those with good practices towards dengue (aOR: 3.39, 95% CI: 1.37–8.38, *p* = 0.008) had better knowledge.

**Conclusion:**

Context-specific prevention messages on dengue and targeted interventions should be developed for IDPs and residents from semi-urban areas in Burkina Faso.

**Contribution:**

This study provides findings to enhance dengue awareness among vulnerable populations and preparedness for future epidemics.

## Introduction

Dengue is a viral infection transmitted to humans through the bite of mosquitoes (*Aedes aegypti* or *Aedes albopictus*).^1,2,3^ When it is symptomatic, the main manifestations are fever, headaches, joint pain, nausea, and vomiting. Dengue is a major public health concern, particularly in tropical and subtropical regions where it re-emerges and occurs throughout endemic and epidemic episodes.^4^ During the past two decades, the spread of dengue has been driven by climate change, human migration, and uncontrolled urbanisation.^5^ In 2024, the World Health Organization (WHO) estimated more than 7.5 million cases of dengue worldwide, resulting in more than 10 000 deaths.^6^ These figures are increasing, from 505 430 cases in 2000 to 5.2 million in 2019, as well as an excess mortality rate of 65% between 2000 and 2017.^4,5^

The West Africa region is among the most affected by dengue. The number of confirmed cases evolved from 14 000 in 2014 to 70 000 in 2023, and Burkina Faso accounted for 85% of all reported cases and 90% of all deaths.^7^ The first cases of dengue in this country were documented in 1925.^8,9^ Since then, the country has officially declared outbreaks in 2006, 2016, and 2023.^10,11^ By the end of the 33rd week of 2023, Burkina Faso had recorded 79 867 suspected cases, 34 687 probable cases, and 349 deaths.^12^ The outbreak mainly affected the two largest cities (Ouagadougou and Bobo-Dioulasso) and other secondary ones. It resulted in high number of hospitalisations, overcrowding in emergency and inpatient services, and disruptions in healthcare provision. In addition, cases of self-medication with herbal remedies among hospitalised patients and nephrological complications such as tubular necrosis were documented.^13,14^

Similar to other vector-borne diseases, the control of dengue is influenced by structural factors, including the organisation of curative and preventive care, environmental factors (such as sanitation), and individual factors (such as the use of repellents and bed nets).^15,16^ In Burkina Faso, the healthcare context has been exacerbated since 2015 by a security crisis, which has led to the displacement of populations from rural to urban and semi-urban areas. Internally displaced populations (IDPs) are particularly vulnerable to health issues, including dengue.^17^ However, only a few studies conducted in Burkina Faso have examined individual factors contributing to the dengue burden, particularly among these vulnerable populations. Specific information on populations’ knowledge, attitudes, and practices (KAP) regarding dengue is essential to better organise the national response, particularly for guiding awareness and prevention messages. We hypothesise that IDPs compared to resident populations have poorer knowledge, less favourable attitudes, and worse practices regarding dengue.

This study aims to assess the knowledge, attitudes, and practices regarding dengue in resident populations and in IDPs settled in Tougan, and to identify factors associated with good knowledge of dengue in 2024.

## Research methods and design

### Study design and setting

We conducted a cross-sectional study between April 2024 and July 2024 in Tougan, a semi-urban city in northwestern Burkina Faso, bordering Mali. Tougan is part of the Boucle du Mouhoun region and had a population of 26 347 in 2022.^18^ In March 2023, the ‘Secrétariat Permanent du Conseil National des Secours d’Urgence et de Réhabilitation’ reported a total of 134 000 IDPs as a result of insecurity in the Boucle du Mouhoun.^19^ During the study period, Tougan comprised seven sectors (or neighbourhoods) and four IDP camps.

### Study population and sampling

The study population was composed of resident and displaced individuals aged 18 years or older, present at the time of the study, and consented to participate. Participants were selected using an adapted three-stage random sampling method. Firstly, three sectors and two IDP camps were randomly chosen. Secondly, households were randomly selected. Thirdly, within each household, an eligible individual, usually the head of the household, was chosen to respond to the questionnaire.^20^ Choosing the head of household is mainly related to norms around decision-making in this context.

A minimum sample size was calculated using Schwartz’s formula.^21^ We assumed a previously documented knowledge proportion of 50% and a first-type error risk (α) of 5%. Accounting for a potential 10% refusal rate, the required sample size was 419 individuals.

### Data collection and study variables

Data were collected using a face-to-face questionnaire administered by trained interviewers. The questionnaire was based on the literature review and included sociodemographic information, as well as knowledge, attitudes, and practices regarding dengue. Knowledge was assessed through 13 questions allowing mutually exclusive answers: ‘yes’, ‘no’, or ‘don’t know’. Correct answers were assigned a score of 1, while incorrect answers were scored 0. The total knowledge score ranged from 0 to 13. Knowledge was classified as poor (score ≤ 7), moderate (score 8–10) or good (score ≥ 11). Attitudes were assessed using eight questions with response options ‘yes’, ‘indifferent’, or ‘no’, scored as 2, 1, and 0, respectively. The total attitude score ranged from 0 to 16 and was classified as unfavourable (≤ 8), acceptable (9–13), or favourable (≥ 14). Practices were assessed using seven questions with response options ‘always’, ‘sometimes’, and ‘never’, scored as 2, 1, and 0, respectively. The total practice score ranged from 0 to 14 and was classified as poor (≤ 7), acceptable (8–10), or good (≥ 11).

### Statistical analysis

Firstly, we described the characteristics of the study participants. Quantitative variables were summarised using means and standard deviations (s.d.), while categorical variables were presented as absolute (counts) and relative (percentages) frequencies. Secondly, we described knowledge, attitudes, and practices regarding dengue and compared them between resident and IDPs using Pearson’s Chi-square test or Fisher’s exact test when appropriate. Thirdly, we identified factors associated with good knowledge of dengue (score ≥ 11) using a logistic regression. Variables associated with good knowledge of dengue with a *p*-value ≤ 0.20 were specified in the complete multivariate model. A manual backward elimination based on the log-likelihood method was used to determine the final multivariate model.

For all calculations, statistical significance was defined with a *p*-value ≤ 0.05. The goodness of fit of the retained model was assessed using the Hosmer and Lemeshow test. All analyses were performed using STATA software (version 16; Stata Corp LP, College Station, Texas, United States).

### Ethical considerations

Ethical clearance to conduct this study was obtained from the Ministry of Health and Public Hygiene, Ministry of Higher Education, Research and Innovation Health Research Committee (Comité d’Ethique pour la Recherche en Santé [CERS] No. 2024-04-119). All participants provided written informed consent. Illiterate participants benefited from the information note translation in their mother tongue by a person that they choose. Anonymity as well as confidentiality were respected during all the study steps (data collection, analysis, and diffusion).

## Results

### Characteristics of the study participants

A total of 419 participants, including 226 (53.9%) men, were included in the study. Their mean age was 38.5 years (s.d.: 12.9 years). A total of 289 (68.9%) participants were residents, while 130 (31.1%) were IDPs. Slightly more than two-thirds of the population (*n* = 289, 68.9%) had an education level below secondary school. Two-thirds of the participants (*n* = 269, 64.2%) engaged in domestic animal husbandry. The characteristics of the participants are presented in Table 1.

**TABLE 1:** Characteristics of the 419 participants.

Variables	All participants (*N* = 419)				Residents (*n* = 289)				IDPs (*n* = 130)				*p*-value
	*n*	%	mean	s.d.	*n*	%	mean	s.d.	*n*	%	mean	s.d.	
**Age in years**	-	-	38.5	12.9	-	-	39.3	12.5	-	-	36.8	12.9	-
18–24	46	11	-	-	22	7.6	-	-	24	18.5	-	-	< 0.001
25–59	326	77.8	-	-	230	79.6	-	-	96	73.8	-	-	0.002
≥ 60	47	11.2	-	-	37	12.8	-	-	10	7.7.	-	-	-
**Sex**													
Male	226	53.9	-	-	148	51.2	-	-	78	60	-	-	0.095
Female	193	46.1	-	-	141	48.8	-	-	52	40	-	-	-
**Education level**													
Never attended school	161	38.4	-	-	95	33	-	-	66	50.8	-	-	0.002
Primary	128	30.5	-	-	97	33.5	-	-	31	23.8	-	-	-
Secondary	130	31.1	-	-	97	33.5	-	-	33	25.4	-	-	-
**Marital status**													
Married or free union	264	63.1	-	-	178	61.6	-	-	86	66.2	-	-	0.371
Single	155	36.9	-	-	111	38.4	-	-	44	33.8	-	-	-
**Occupation**													
Government employee	25	6	-	-	25	8.7	-	-	-	-	-	-	0.001
Liberal	78	18.6	-	-	57	19.7	-	-	21	16.2	-	-	-
Unemployed	316	75.4	-	-	207	71.6	-	-	109	83.8	-	-	-
**Household size (Number of persons)**													
1–5	152	36.3	-	-	111	38.4	-	-	41	31.5	-	-	0.389
6–10	223	53.2	-	-	148	51.2	-	-	75	57.7	-	-	-
≥ 10	44	10.5	-	-	30	10.4	-	-	14	10.8	-	-	-
**Raising domestic animals**													
Yes	269	64.2	-	-	196	67.8	-	-	73	56.2	-	-	0.021
No	150	35.8	-	-	93	32.2	-	-	57	43.8	-	-	-
**Stagnant water**													
Yes	278	66.4	-	-	158	54.7	-	-	120	92.3	-	-	< 0.001
No	141	33.6	-	-	131	45.3	-	-	10	7.7	-	-	-

IDPs, internally displaced populations; s.d., standard deviation.

### Knowledge of dengue

**FIGURE 1:**
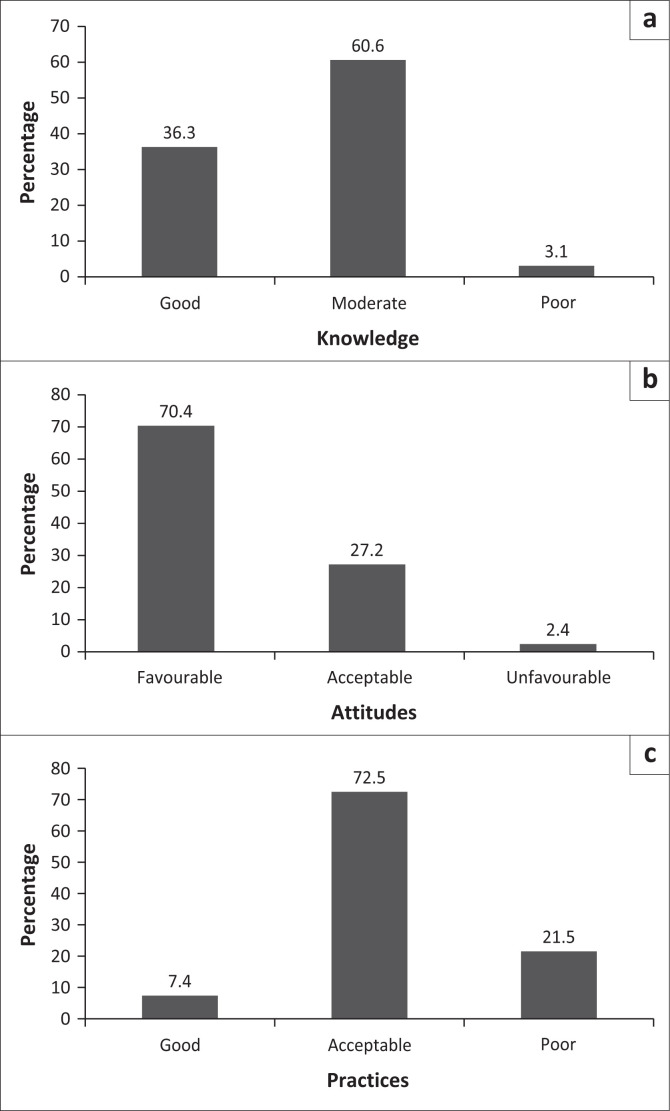
Overall knowledge (a), attitudes (b), and practices (c) of Tougan population towards dengue in 2024 (*N* = 419).

The mean dengue knowledge score was 10.1 (s.d.: 1.2) out of 13. Approximately one-third of the participants (*n* = 152/419, 36.3%) had good knowledge, more than half (*n* = 254/419, 60.6%) had moderate knowledge, and 13 out of 419 (3.1%) had poor knowledge (Figure 1). Half of the participants (49.9%) believed that there was no difference between malaria and dengue. Specifically, IDPs confused the two diseases more than resident populations (74.6% vs. 38.6%, *p* < 0.001). Overall knowledge of dengue symptoms and prevention methods was 98.8% and 100%, respectively. These figures did not differ significantly according to population type (99.6% vs. 96.9%, *p* = 0.075, and 100% vs. 100%, *p* = 0.574, respectively). Details of responses to the knowledge-related questions are presented in Table 2.

**TABLE 2:** Distribution of the 419 participants according to their knowledge of dengue.

Questions	All participants (*N* = 419)		Residents (*n* = 289)		IDPs (*n* = 130)		*p*-value
	*n*	%	*n*	%	*n*	%	
**Are dengue and malaria the same disease?**							
Yes	209	49.9	112	38.8	97	74.6	< 0.001
No†	210	50.1	177	61.2	33	25.4	-
**Is it possible to be infected with dengue fever more than once?**							
Yes†	327	78.1	208	71.9	119	91.5	< 0.001
No	92	21.9	81	28.1	11	8.5	-
**Is dengue a fatal disease?**							
Yes†	407	97.2	277	95.8	130	100	0.07
No	12	2.8	12	4.2	0	-	-
**Can dengue affect children as well as adults?**							
Yes†	392	93.6	262	90.6	130	100	< 0.001
No	27	6.4	27	9.4	0	-	-
**Is the mosquito the insect that transmits dengue fever?**							
Yes†	415	99	289	100	126	96.9	0.135
No	4	10	0	-	4	3.1	-
**Is the rainy season a period of high dengue transmission?**							
Yes†	419	100	289	100	130	100	-
No	0	-	0	-	0	-	-
**Can dengue infect a newborn baby?**							
Yes†	310	74	195	67.6	115	88.4	< 0.001
No	109	26	94	32.4	15	11.6	-
**Can dengue infect a pregnant woman?**							
Yes†	387	92.4	270	93.4	117	90	< 0.001
No	32	7.6	19	6.6	13	10	-
**Is there a specific medical treatment for dengue?**							
Yes	349	83.3	237	82.1	112	86.2	< 0.001
No†	70	16.7	52	17.9	18	13.8	-
**Can dengue fever be cured using modern medicines (hospital)?**							
Yes†	343	81.9	232	80.2	111	85.4	0.027
No	76	18.1	57	19.8	18	13.8	-
**Is there a vaccine against dengue fever?**							
Yes†	108	25.8	99	34.3	9	6.9	< 0.001
No	311	74.2	190	65.7	121	93.1	-
**Are the following symptoms suggestive of dengue fever?** ‡							
Oui†	414	98.8	288	99.6	126	96.9	0.075
Non-	4	1.2	1	0.4	4	3.1	-
**Do the following preventive measures protect against dengue fever?§**							
Oui†	419	100	289	100	130	100	0.574
Non	0	-	0	-	0	-	-

† , Expected correct answer;

‡ , Headaches, retro-orbital pain, feeling of hunger, diarrhoea, fatigue;

§ , Facial mask, repellents, bed nets, hand washing, draining stagnant water.

### Attitudes towards dengue

The mean attitude score towards dengue was 13.5 (s.d.: 1.9) out of 16. A total of 295 participants (70.4%) had favourable attitudes, 114 (27.2%) had acceptable attitudes, and 10 (2.4%) had unfavourable attitudes (Figure 1). Attitudes towards dengue were significantly more favourable in IDPs than in resident populations (85.4% vs. 63.7%, *p* < 0.001). Details of responses to the attitudes related questions are presented in Appendix 1 – Table 1-A1.

### Practices regarding dengue

The mean dengue practice score was 9.4 (s.d.: 2.2) out of 14. Very few participants (*n* = 31, 7.4%) had good dengue-related practices, 304 (72.6%) had moderate practices, and 84 (20.1%) had poor practices (Figure 1). Good practices with regard to dengue were significantly higher in resident populations than in IDPs (9.3% vs. 3.1%, *p* < 0.001). Details of responses to the practices related questions are presented in Appendix 1 – Table 2-A1.

### Factors associated with good knowledge of dengue

The level of knowledge in IDPs was significantly lower than that in residents (adjusted odds ratio [aOR]: 0.49, 95% confidence interval [CI]: 0.29–0.81, *p* = 0.006) (Table 3). Participants who reported stagnant water in their homes had a higher level of knowledge (aOR: 3.03, 95% CI: 1.84–4.96, *p* < 0.001) than those who did not. Participants who reported good practices towards dengue also had better knowledge (aOR: 3.39, 95% CI: 1.37–8.38, *p* = 0.008) compared to those who did not.

**TABLE 3:** Factors associated with Tougan population’s knowledge of dengue in 2024 (Logistic regression models).

Variables	Good knowledge		Bad knowledge		Crude		*p*-value	Adjusted		*p*-value
	*n*	%	*n*	%	OR	95% CI		OR	95% CI	
**Type of population**										
IDPs	37	28.5	93	71.5	0.6	0.38–0.94	0.026	0.49	0.29–0.81	0.006
Resident	115	39.8	174	60.2	1	-	-	-	-	-
**Age in years**										
18–24	13	28.3	33	71.7	1	-	-	-	-	-
25–59	119	36.5	207	63.5	1.45	0.73–2.88	0.276	-	-	-
≥ 60	20	42.5	27	57.5	1.88	0.79–4.46	0.152	-	-	-
**Sex**										
Male	87	38.5	139	61.5	1.23	0.82–1.84	0.307	-	-	-
Female	65	33.7	128	66.3	1	-	-	-	-	-
**Education**										
Never attended school	61	37.9	100	62.1	1	-	-	-	-	-
Primary	49	38.3	79	61.7	1.01	0.63–1.63	0.946	-	-	-
Secondary	42	32.3	88	67.7	0.78	0.48–1.27	0.323	-	-	-
**Marital status**										
Célibataire	59	38.1	96	61.9	1	-	-	-	-	-
Married or in a couple	93	35.2	171	64.8	0.88	0.58–1.33	0.56	-	-	-
**Occupation**										
Government employed	1	40	15	60	1	-	-	-	-	-
Liberal	28	35.9	50	64.1	0.84	0.33–2.11	0.712	-	-	-
Unemployed	114	36.1	202	63.9	0.84	0.36–1.94	0.695	-	-	-
**Domestic animals**										
Yes	101	37.5	168	62.5	1.16	0.76–1.77	0.469	-	-	-
No	51	34	99	66	1	-	-	-	-	-
**Household size**										
1–5	58	38.2	94	61.8	1	-	-	-	-	-
6–10	77	34.5	146	65.5	0.85	0.55–1.31	0.472	-	-	-
≥ 10	17	38.6	27	61.4	1.02	0.51–2.03	0.954	-	-	-
**Stagnant water**										
Yes	116	41.7	162	58.3	2.08	1.33–3.26	0.001	3.03	1.84–4.96	< 0.001
No	41	29.1	100	70.9	1	-	-	-	-	-
**Practices**										
Good	18	58.1	13	41.9	3.67	1.55–8.67	0.003	3.39	1.37–8.38	0.008
Acceptable	111	36.5	193	63.5	1.52	0.89–2.60	0.121	-	-	-
Poor	23	27.4	61	72.6	1	-	-	-	-	-

IDP, internally displaced populations.

## Discussion

Our study highlighted a low level of knowledge of dengue in the population of Tougan, acceptable attitudes, and poor practices. The IDPs had lower knowledge and poorer practices than resident populations. However, they had better attitudes.

### Knowledge, attitudes and practices towards dengue

Although IDPs had lower levels of knowledge than residents, it is noteworthy that overall knowledge remained low. Our findings are consistent with a study conducted in Ouagadougou in 2015, which also found a low level of knowledge of dengue.^22^ This may be partly explained by the fact that dengue is a relatively new public health issue in Burkina Faso and is therefore not widely known, especially in secondary cities such as Tougan. These results highlight the need for educational efforts to enhance dengue-related knowledge among the population of Burkina Faso, particularly in rural and semi-urban areas.

A thorough analysis of the responses to knowledge-related questions revealed three key points requiring focused communication to dispel misconceptions and establish accurate information. The first point is the confusion between malaria and dengue. Half of the respondents believed that there was no difference between these two diseases. This confusion may arise from the similarities in their clinical manifestations (headache, fever, joint and muscle pain) and the fact that both are mosquito-borne illnesses. Moreover, it is important to observe that dengue infection is commonly called as ‘paludengue’ by the Burkina Faso population.

The second point requiring clarification is the availability of dengue treatment. Only 16% of respondents knew that dengue has no specific treatment. This is crucial, as a significant proportion of participants reported using traditional medicine when experiencing dengue-like symptoms. Previous studies in Burkina Faso documented an acute kidney failure rate of 20% among patients hospitalised for severe dengue during the 2016 outbreak.^11^ This complication was partially attributed to self-medication and the use of traditional treatments, often decoctions of roots and other plant parts.

The third point for communication and intervention is the existence of a dengue vaccine and its potential future implementation.^23,24^ In May 2024, the WHO recommended the use of recombinant live-attenuated vaccines (CYD-TVD and TAK-003) for dengue prevention. The implementation of these vaccines in Burkina Faso, particularly among vulnerable populations, could help prevent future outbreaks.

Despite the existence of several knowledge gaps, responses regarding symptoms and prevention methods were largely accurate. This encouraging result may be because of the population’s association of dengue with malaria. Strengthening this knowledge by clearly distinguishing the similarities and differences between these two diseases is essential.

Overall, participants had favourable attitudes towards dengue, with IDPs demonstrating more positive attitudes than resident populations. This is encouraging, as it suggests that the population is willing to take appropriate preventive and management actions. National dengue response efforts should capitalise on this positive dynamic when designing interventions.

However, dengue-related practices remained poor, particularly among IDPs. This result may be explained by the suboptimal knowledge levels. Intuitively, an individual’s practices in a given domain are often determined by their level of knowledge in the same area.^25^ This is the fundamental principle behind behaviour change education. In addition, IDPs often live in challenging social conditions, sometimes lacking proper housing, which may make issues like environmental sanitation a lower priority.

### Study’s limitations

Our findings should be interpreted with caution. Firstly, the study was conducted in a single site, the semi-urban town of Tougan, making it difficult to generalise results to other semi-urban areas in Burkina Faso. Secondly, the fact of choosing sometimes the heads of households at the third stage of population selection could result in a potential bias. Thirdly, the data had been collected using an invalid tool (questionnaire). Fourthly, given the face-to-face data collection method, social desirability bias may have influenced some responses, potentially leading to an overestimation of good practices. Fifthly, certain key potential confounders for good knowledge on dengue (e.g. access to health information, duration of displacement) were not collected. Nevertheless, our study is one of the first in these vulnerable populations in Burkina Faso. The findings will help filling an important data gap regarding dengue-related KAPs, designing effective prevention messages, and improving dengue preparedness.

## Conclusion

The population of Tougan has insufficient specific knowledge about dengue. Moreover, differences in knowledge, attitudes, and practices were observed between residents and IDPs. There is an urgent need to develop and disseminate context-specific prevention messages and targeted interventions to these populations in semi-urban areas in Burkina Faso.

## Appendix 1

**TABLE 1-A1:** Distribution of the 419 participants according to their attitudes towards dengue.

Questions	All participants (*N* = 419)		Residents (*n* = 289)		IDPs (*n* = 130)		*p*-value
	*n*	%	*n*	%	*n*	%	
**If you were ill with dengue fever, would you be willing to go to a health facility for treatment?**							
Yes	415	99	287	99.3	128	98.4	-
No	2	0.5	2	0.7	0	00.0	0.409
No interest	2	0.5	0	0	2	1.6	-
**If you were ill with dengue fever, would you be willing to use traditional medicines for your treatment?**							
Yes	300	71.6	176	60.9	124	95.4	0.000
No	99	23.6	97	33.5	4	3.1	-
No interest	20	4.8	16	5.6	2	1.5	-
**Would you be willing to rest or sleep in the same place as someone suffering from dengue?**							
Yes	329	78.5	216	74.8	113	86.9	0.002
No	58	13.8	56	19.4	2	1.6	-
No interest	32	7.7	17	5.8	15	11.5	-
**Would you be willing to sleep under an insecticide-treated bed net to avoid dengue fever?**							
Yes	393	93.8	265	91.7	128	98/4-	-
No	20	4.8	20	6.9	0	0	0.004
No interest	6	1.4	4	1.4	2	1.6	-
**Would you be willing to eat on the same plate as someone suffering from dengue fever?**							
Yes	354	84.5	239	82.7	115	88.4	0.196
No	31	7.4	29	10.1	2	1.6	-
No interest	34	8.1	21	7.2	13	10	-
**Would you be willing to use repellents (ointments or insecticides) to avoid dengue fever?**							
Yes	384	91.6	256	88.5	128	98.4	0.011
No	23	5.5	23	7.9	0	0	-
No interest	12	2.9	10	3.6	2	1.6	-
**Would you be willing to clean up your living environment (dry out water jets-, keep rubbish bins covered) to avoid dengue fever?**							
Yes	396	94.5	267	92.4	129	99.2	0.116
No	19	4.5	19	6.5	0	0	-
No interest	4	1	3	1.1	1	0.8	-
**Would you be willing to attend a sensitising dengue awareness programme?**							
Yes	402	95.9	273	94.5	129	99.2	0.016
No	14	3.4	14	4.8	0	0	-
No interest	3	0.7	2	0.7	1	0.8	-

IDP, internally displaced population.

**TABLE 2-A1:** Distribution of the 419 participants according to their practices towards dengue.

Questions	All participants (*N* = 419)		Residents (*n* = 289)		IDPs (*n* = 130)		*p*-value
	*n*	%	*n*	%	*n*	%	
**Do you use traditional medicines (decoctions) when you or a member of your family is experiencing symptoms suggestive of dengue fever?**							
Always	230	54.9	126	43.6	104	80	-
Not always	96	22.9	73	25.3	23	17.1	0.002
Never	93	22.2	90	31.1	3	2.4	-
**Do you refer in priority to a health facility when you or a member of your family is ill (or has signs suggestive of dengue fever)?**							
Always	212	50.6	168	58.1	44	33.8	-
Not always	205	48.9	119	41.2	86	66.2	0.18
Never	2	0.5	2	0.7	0	0	-
**Do you use body repellents against mosquitoes, especially during the rainy season?**							
Always	141	33.7	101	35	40	30.7	0.069
Not always	184	43.9	128	44.2	56	43.1	-
Never	94	22.4	60	0.7	34	26.2	-
**Do you sleep under insecticide-treated bed nets during the rainy season?**							
Always	234	55.8	199	68.8	35	26.9	0.169
Not always	136	32.5	55	19.1	81	62.3	-
Never	49	11.7	35	12.1	14	10.8	-
**Do you wear long clothes during the rainy season to protect yourself from mosquito bites?**							
Always	140	33.4	120	41.5	20	15.4	0.016
Not always	266	63.5	157	54.3	109	83.8	-
Never	13	3.1	12	4.2	1	0.8	-
**Do you think you clean up your living environment enough during the rainy season?**							
Always	236	56.3	190	65.8	46	35.4	-
Not always	160	38.2	83	28.7	77	59.2	0.029
Never	23	5.5	16	5.5	7	5.4	-
**Do you regularly check your home and its surroundings for seeking mosquito breeding sites?**							
Always	172	41	163	56.4	9	6.9	-
Not always	157	37.5	103	35.7	54	41.5	0.018
Never	90	21.5	23	7.9	67	51.6	-

IDP, internally displaced population.
